# Preoperative mild cognitive impairment as a risk factor of postoperative cognitive dysfunction in elderly patients undergoing spine surgery

**DOI:** 10.3389/fnagi.2024.1292942

**Published:** 2024-01-12

**Authors:** Sujung Park, Jeongmin Kim, Yoon Ha, Keung N. Kim, Seong Yi, Bon-Nyeo Koo

**Affiliations:** ^1^Department of Anesthesiology and Pain Medicine, Yonsei University College of Medicine, Seoul, Republic of Korea; ^2^Anesthesia and Pain Research Institute, Yonsei University College of Medicine, Seoul, Republic of Korea; ^3^Department of Neurosurgery, Yonsei University College of Medicine, Seoul, Republic of Korea; ^4^POSTECH Biotech Center, Pohang University of Science and Technology, Pohang, Republic of Korea

**Keywords:** neurocognitive disorders, perioperative care, postoperative cognitive complications, cognitive dysfunction, aged, delirium

## Abstract

**Introduction:**

Any persistent degree of cognitive impairment in older adults is a concern as it can progress to dementia. This study aimed to determine the incidence and risk factors for early postoperative cognitive dysfunction (POCD) in elderly patients undergoing spine surgery.

**Methods:**

Patients were enrolled from a previous prospective observational study after screening for normal cognitive function using the Mini Mental State Examination (MMSE). Cognitive function was evaluated before surgery and at 1 week, month, and year post-surgery using MMSE and Montreal Cognitive Assessment scores (MoCA). Mild cognitive impairment (MCI) was determined using the MoCA scores adjusted for age. POCD was defined as a drop of three or more points on the MMSE 1 week post-surgery. Multivariate logistic analysis was performed to identify POCD risk factors.

**Results:**

A total of 427 patients were included. Eighty-five (20%) had pre-existing MCI. The MCI group showed lower MoCA scores at each time point (baseline, 1 week after surgery, 1 month after surgery, 1 year after surgery) compared to the non-MCI group. Those in the MCI group had a higher rate of admission to intensive care unit after surgery, postoperative delirium, and POCD 1 week post-surgery, than those in the non-MCI group (16.5% vs. 6.7%, *p* = 0.008; 27.1% vs. 15.8%, *p* = 0.024; and 18.8% vs. 8.2%, *p* < 0.001, respectively). Among them, 10.3% were assessed for POCD on postoperative day 7 and self-reported poor social roles and physical functioning 1 week postoperatively.

**Conclusion:**

Preoperative MCI was seen in ~20% of surgical patients aged >70 years. POCD was seen in ~20% of patients with pre-existing MCI, and ~ 10% of those without. Benzodiazepine use, significant comorbidities, pre-existing MCI, and depressive tendencies were risk factors for POCD.

## 1 Introduction

With advances in medical technology, the number of elderly patients undergoing surgery continues to increase (Lee et al., 2015). It is generally anticipated that cognitive function will decline as part of the normal aging process. MCI is often considered an early stage of dementia or a transitional phase between the normal aging process and dementia. MCI is characterized by cognitive changes that are greater than expected for one’s age but do not prominently impair daily function (Arevalo-Rodriguez et al., 2021). The estimated prevalence of MCI is 10–66% in elderly patients (Petersen, 2011; Langa and Levine, 2014; Schrempft et al., 2022; Lee et al., 2023). The lack of obvious criteria for the diagnosis of MCI and variations in population characteristics among different studies have resulted in considerable variation in prevalence among established research. MCI increases healthcare utilization, caregiver burden, development of delirium, and even mortality (Petersen, 2011; Culley et al., 2017; Bae et al., 2018). The importance of evaluating cognitive function before surgery for preoperative MCI has been emphasized (Culley et al., 2017); however, in actual clinical situation, a preoperative cognitive examination is not routinely performed for all elderly patients scheduled for surgery.

Postoperative cognitive dysfunction (POCD) is typically characterized as a type of cognitive impairment that emerges within a timeframe ranging from 7 days to 1 year following the surgical procedure (Needham et al., 2017). POCD is generally temporary but may be linked to delays in returning to work, a higher occurrence of long-term dementia, and even mortality (Steinmetz et al., 2009; Bilotta et al., 2010; Lin et al., 2022). POCD is a major concern in patients undergoing surgery, and a few reports have indicated that the symptoms of cognitive decline may persist long after surgery (Steinmetz et al., 2009; Leslie, 2017).

This study aimed to identify the incidence and risk factors of early POCD in elderly patients with normal cognitive function undergoing spine surgery. We hypothesized that pre-existing cognitive impairment is associated with POCD.

## 2 Materials and methods

### 2.1 Ethics approval

This was a secondary analysis of a previous prospective observational study that primarily focused on postoperative delirium and was approved by the local institutional review board (Severance Hospital 4–2019-0654; ClinicalTrials.gov Identifier: NCT04120272). Written informed consent was obtained from all participants.

### 2.2 Participants

We screened patients aged >70 years scheduled to undergo spine surgery between November 2019 and May 2023. We screened patients within the week before surgery using the MMSE, finally enrolling 600 patients with normal cognition. The exclusion criteria were presence of literacy problems, language difficulties, and hearing or visual impairment and a history of neurologic disease (e.g., stroke, seizures, dementia, and cognitive impairment). Medical comorbidities, medical history, and years of education were recorded through meticulous searches of medical records and interviews with patients and caretakers.

The MMSE is a concise assessment tool that evaluates various aspects of cognitive function such as orientation, memory, language, attention, calculation, praxis, and visuospatial function. With a maximum score of 30, a higher score represents better cognitive function. The Montreal Cognitive Assessment (MoCA) is a screening tool that was developed specifically for the detection of MCI and takes approximately 10 min to complete. Many studies that directly compared the MoCA and MMSE have revealed that the MoCA is more sensitive in accurately distinguishing individuals with MCI from those with normal cognitive function (Roalf et al., 2013; Langa and Levine, 2014). The MMSE and MoCA were administered by trained interviewers four times: baseline tests within the week before surgery, on postoperative day 7, and at 1 month and 1 year after surgery. The modified version of the Telephone Interviews for Cognitive Status (TICS-m) was implemented to minimize the rate of loss to follow-up for discharged patients, both at the 1 month and 1 year postoperative periods. Depression was assessed using the Geriatric Depression Scale (GDS). The GDS, originally developed by Yesavage et al. (1982), is a self-reporting instrument used extensively for the comprehensive geriatric assessment of depression (Yesavage et al., 1982). The total score is the sum of the scores for the individual questions (maximum score of 30). The PROMIS (Patient-Reported Outcomes Measurement Information System)-29 V2.1 consists of a set of patient-centered measures: sleep disturbance, social roles, physical function, fatigue, anxiety, and depression (Kang et al., 2021; Kim et al., 2021). We used a five-point Likert scale (range: 1–5) to measure the severity or frequency of symptoms. Higher scores indicated improved physical function; increased engagement in social roles and activities; and higher levels of anxiety, depression, fatigue, and sleep disruption. The scores in each domain were converted into T-scores using the PROMIS scoring algorithms. The *T*-scores have a mean of 50 and a standard deviation of 10 in the general population, allowing for comparisons across different populations. The use of the PROMIS for assessing the quality of life of patients with cancer or surgery is becoming increasingly popular (Hatchimonji et al., 2021; Kenfack et al., 2022).

### 2.3 Definition of POCD, postoperative delirium, and MCI

Pre-existing MCI was assessed before surgery. Age-adjusted MoCA cut-off scores for MCI detection were obtained from a study in the Korean population (Kang et al., 2009; Kwak and Kim, 2021). The cut-off scores were < 22 for 70–79-year-olds, < 18 for 80–84-year-olds, and < 14 for 85–89-year-olds. Postoperative delirium was assessed twice daily by trained study personnel from the day of surgery until postoperative day 7 in the CAM-ICU. POCD was defined as a decrease of three points compared with the baseline MMSE score 1 week after surgery (Wu and Han, 2020; Wang et al., 2021; Xi et al., 2021; Huang et al., 2022).

### 2.4 Anesthetic management

All surgical procedures and anesthetic management were performed using standardized maneuvers at our institute. The procedures included laminectomy, discectomy, and spinal fusion and were performed with the patient in the prone position and the head and neck in the neutral position.

Anesthesia was induced with propofol (1–1.5 mg/kg IV), remifentanil (0.05–0.2 μg/kg^/^min), and rocuronium (0.6 mg/kg) and maintained with sevoflurane or desflurane. Automated measurements and recordings of blood pressure, heart rate, and peripheral oxygen saturation were performed continuously. During surgery, the concentration of sevoflurane or desflurane was adjusted according to the patient’s state index (Psi), using a SedLine^®^ sensor (Masimo, Irvine, CA, United States). Intraoperative SedLine^®^ analysis was performed on 241 patients.

### 2.5 Study outcomes

The primary outcome was POCD incidence 1 week after surgery. The secondary outcomes were MCI incidence among normal elderly patients; risk factors for POCD; and MMSE, MoCA, TICS-m, or PROMIS-29 scores at several time point such as preoperative period (baseline), 1 week after surgery, 1 month after surgery and 1 year after surgery.

### 2.6 Statistical analysis

The Shapiro–Wilk test was used to assess the normality of the variables. We used the independent Student’s *t*-test for normally distributed and the Wilcoxon rank-sum test for non-normally distributed variables. Normal and non-normal variables are represented as mean ± SD and median (IQR), respectively. The chi-squared test was used for categorical variables expressed as numbers (%). In the results of cognitive function test (MMSE, MoCA), the comparison between the group according to the 4 time points (baseline, 1 week, 1 month, 1 year) were analyzed using a linear mixed-effect model. Post-hoc tests were conducted using the emmeans package. Multivariate logistic analysis was performed to identify the risk factors for POCD in elderly patients. Certain factors such as age, level of education, cerebrovascular disease, duration of operation, poor functional status, time spent with low Psi, and pre-existing cognitive impairment have been identified as risk factors for POCD in previous studies (Needham et al., 2017; Tasbihgou and Absalom, 2021). Thus, these factors were considered as potential confounding variables in the present analyses. Data manipulation and analyses were performed using the R software, version 4.1.0 (R Foundation for Statistical Computing, Vienna, Austria).

## 3 Results

**Figure 1:**
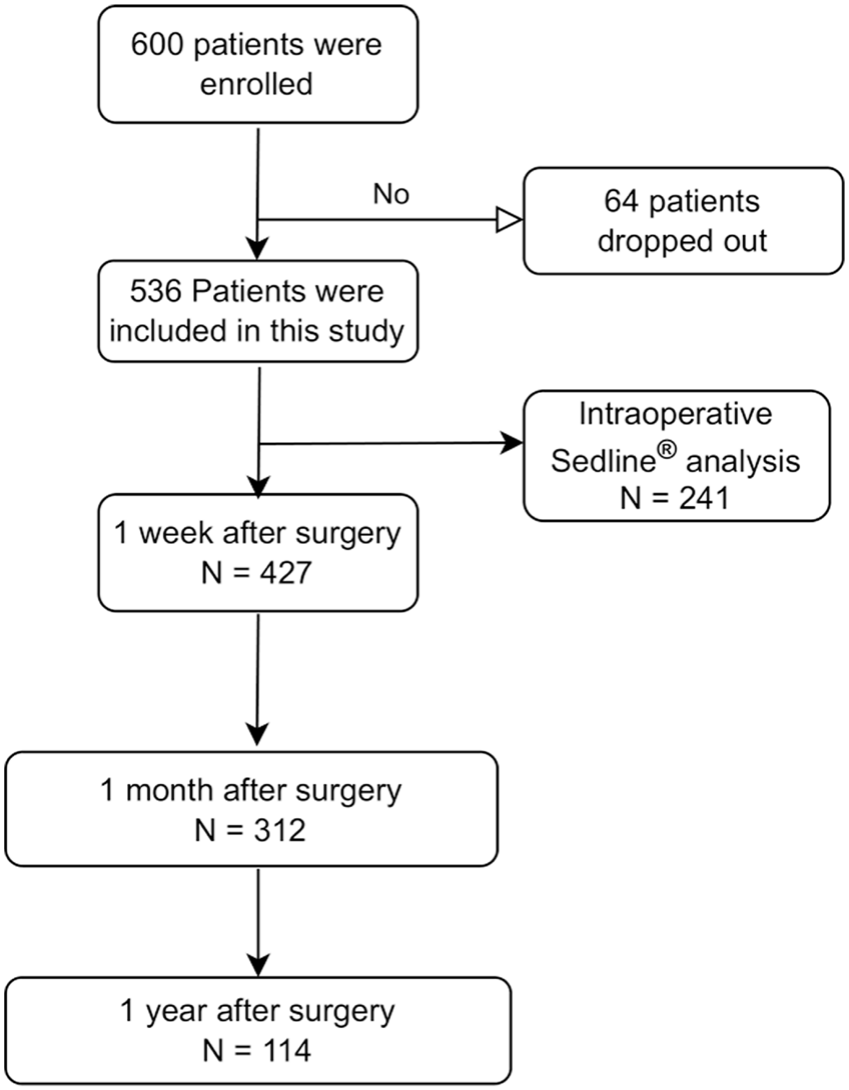
Patient inclusion flow chart.

A total of 600 patients were enrolled, and 64 dropped out owing to cancelation of the planned surgery and/or withdrawal of consent. Thus, 536 patients completed the study, and 427 patients who completed the preoperative MMSE, preoperative MoCA, and MMSE 1 week after surgery were finally analyzed (Figure 1).

In this study, preoperative MCI was suspected in many patients (19.9%, 85/427), despite excluding those with cognitive impairment and dementia using the baseline MMSE test, based on years of education. Patients with preoperative cognitive impairment had a higher percentage of being female and having poorer functional status, higher scores on the frailty index and GDS, and lower education level (Table 1).

**Table 1:** Baseline characteristics of MCI and non-MCI groups.

	MCI	Non-MCI	*p*-value
	(*N* = 85)	(*N* = 342)	
Sex (M/F)	14/71 (16.5%)	135/207 (39.5%)	<0.001
Age (year)	74.0 (71.0,77.0)	75.0 (72.0, 78.0)	0.284
Height (cm)	153.0 (150.0, 159.0)	157.3 (151.1, 164.0)	<0.001
Weight (kg)	59.0 (54.0, 64.0)	60.0 (55.0, 67.0)	0.116
BMI	24.8 (22.8, 26.8)	24.2 (22.6, 26.1)	0.273
Frailty index	2.0 (1.0, 3.0)	2.0 (1.0, 2.0)	0.013
Charlson comorbidity index	4.0 (3.0, 4.0)	4.0 (3.0, 4.0)	0.652
Geriatric depression scale	4.0 (1.0, 8.0)	3.0 (1.0, 6.0)	0.048
Education			<0.001
0–3 year	30 (35.3%)	11 (3.2%)	
4–6 year	38 (44.7%)	109 (31.9%)	
7–12 year	15 (17.6%)	162 (47.4%)	
Over 13 years	2 (2.4%)	60 (17.5%)	
history of past delirium	6 (7.1%)	10 (2.9%)	0.141
Benzodiazepine medication	8 (9.5%)	25 (7.4%)	0.683

MCI was diagnosed using age-adjusted cutoff scores of the MoCA score tested within 1 week before surgery (<22 for 70–79-year-olds, <18 for 80–84-year-olds, and < 14 for 85–89-year-olds). Values are presented as the median (Q1, Q3) or number of patients (%). MCI, mild cognitive impairment; BMI, body mass index; MoCA, The Montreal Cognitive Assessment.

The intraoperative parameters were comparable between the MCI and non-MCI groups (Table 2). However, the duration of low Psi under general anesthesia (*T*_Psi < 25_) was higher in the MCI than the non-MCI group. More patients were admitted to the intensive care unit after the surgery in the MCI group compared to the non-MCI group (16.5% vs. 6.7%, *p* = 0.008). The incidence of postoperative delirium was higher in the MCI than the non-MCI group (27.1% vs. 15.8%, *p* = 0.024, Table 2).

**Table 2:** Perioperative parameters and postoperative outcomes in the MCI and non-MCI groups.

	MCI	Non-MCI	*p*-value
Anesthesia duration	220.0 (170.0, 275.0)	215.0 (180.0, 270.0)	0.524
Intraoperative bleeding	500.0 (200.0, 1050.0)	500.0 (200.0, 850.0)	0.572
Lowest heart rate during the operation	55.0 (51.0, 62.0)	56.0 (51.0, 63.0)	0.431
Lowest mean blood pressure during the operation	64.0 (61.0, 70.0)	65.0 (62.0, 69.0)	0.632
*T*_Psi < 25_^*^	8.6 (0.0, 103.0)	0.7 (0.0, 36.2)	0.042
ICU admission	14 (16.5%)	23 (6.7%)	0.008
Postoperative hospital day	8.8 ± 9.3	8.1 ± 5.9	0.559
Incidence of delirium	23 (27.1%)	54 (15.8%)	0.024
Duration of delirium	48.0 (24.0, 84.0)	48.0 (24.0, 96.0)	0.981
Subtype of delirium			0.103
Hyper	8 (34.8%)	26 (48.1%)	
Hypo	2 (8.7%)	11 (20.4%)	
Mixed	13 (56.5%)	17 (31.5%)	

MCI was diagnosed using age-adjusted cutoff scores of the MoCA score tested within 1 week before surgery (<22 for 70–79-year-olds, <18 for 80–84-year-olds, and < 14 for 85–89-year-olds).

* *T*_Psi < 25_ indicates the time at which Psi was less than 25. Values are presented as mean ± SD, median (Q1, Q3) or number of the patients (%).

MCI, mild cognitive impairment; ICU, intensive care unit; MoCA, The Montreal Cognitive Assessment; SD, standard deviation.

Patients with MCI showed lower MMSE, MoCA, and TICS-m scores up to one year after surgery, indicating that cognitive impairment could persist after surgery (Table 3). MCI patients steadily self-reported higher anxiety levels from the preoperative phase to 1 month after surgery, as determined based on PROMIS scores (Supplementary Table 1).

**Table 3:** Comparison of test scores for cognitive function and patient-centered quality of life at baseline and 1 week, 1 month, and 1 year after surgery between the MCI and non-MCI groups.

	MCI	Non-MCI	*p*-value
MMSE			
Baseline	25.0 (24.0, 27.0)	28.0 (27.0, 29.0)	<0.001*
1 week	24.0 (22.0, 26.0)	28.0 (26.0, 29.0)	<0.001*
POCD^*^	16 (18.8%)	28 (8.2%)	<0.001
1 month (*N* = 145)	27.0 (25.0, 28.0)	29.0 (27.0, 29.0)	<0.001^*^
1 year (*N* = 64)	26.0 (24.0, 28.0)	29.0 (28.0, 30.0)	<0.001^*^
MoCA			
Baseline	19.0 (17.0, 20.0)	25.0 (24.0, 27.0)	<0.001^*^
1 week (*N* = 397)	19.0 (16.0, 21.0)	25.0 (23.0, 27.0)	<0.001^*^
1 month (*N* = 136)	21.0 (18.5, 24.0)	26.0 (24.0, 27.0)	<0.001^*^
1 year	20.4 ± 4.7	26.6 ± 2.3	0.001^*^
Modified version of the telephone interviews for cognitive status (TICS-m)			
1 month (*N* = 137)	29.3 ± 6.2	34.7 ± 5.0	<0.001
1 year (*N* = 114)	29.5 ± 5.6	37.0 ± 5.4	<0.001

* POCD was defined as a decrease of three points compared with the baseline MMSE score 1 week after surgery. Values are presented as mean ± SD, median (Q1, Q3) or number of the patients (%). *p-value for the *post hoc* analysis after the linear mixed model. MCI, mild cognitive impairment; POCD, postoperative cognitive dysfunction; MMSE, Mini Mental State Examination; MoCA, The Montreal Cognitive Assessment; TICS-m.

Among the 427 patients aged over 70 years, the prevalence of POCD based on MMSE scores at 1 week post-surgery was 10.3%. The prevalence of POCD at 1 week post-surgery in the MCI group was higher than that in the non-MCI group (18.8% vs. 8.2%, *p* < 0.001, Table 3). In the group with Mild Cognitive Impairment (MCI), the average MoCA score one year after the surgery was 20.4 ± 4.7, while the non-MCI group had a score of 26.6 ± 2.3 points. This signifies that cognitive dysfunction persists in the MCI group even up to one year post-surgery. Patients in POCD group were observed to have a higher intake of benzodiazepines in their home medication and a preoperative history of delirium (Table 4). Patients with POCD also presented with an intraoperatively longer duration of Psi < 25, and a higher incidence and longer duration of postoperative delirium (Table 5). Patients with POCD self-reported a higher rate of sleep disturbance in the preoperative period than did patients without POCD (Supplementary Table 2). One month after surgery, patients with POCD appeared cognitively impaired based on their MMSE and MoCA scores (Table 6). Patients with POCD reported poor social roles and physical function 1 week after surgery (Supplementary Table 2).

**Table 4:** Baseline characteristics of the POCD and non-POCD groups.

	POCD	Non-POCD	*p*-value
	(*N* = 44)	(*N* = 383)	
Sex (M/F)	9/35 (20.5%)	140/243 (36.6%)	0.051
Age (year)	76.0 (72.5, 79.5)	74.0 (72.0, 77.0)	0.136
BMI	24.1 (21.5, 26.7)	24.2 (22.7, 26.3)	0.606
Frailty index	2.0 (1.0, 3.0)	2.0 (1.0, 2.0)	0.049
Charlson comorbidity index	4.0 (4.0, 5.0)	4.0 (3.0, 4.0)	0.001
Geriatric depression scale	5.5 (2.0, 9.0)	3.0 (1.0, 6.0)	0.001
Education			0.048
0–3 year	7 (15.9%)	34 (8.9%)	
4–6 year	21 (47.7%)	126 (32.9%)	
7–12 year	12 (27.3%)	165 (43.1%)	
Over 13 years	4 (9.1%)	58 (15.1%)	
History of past delirium	6 (13.6%)	10 (2.6%)	0.001
Benzodiazepine medication	9 (20.5%)	24 (6.4%)	0.003

Values are presented as the median (Q1, Q3) or number of patients (%). POCD, postoperative cognitive dysfunction; BMI, body mass index; MMSE, Mini Mental State Examination.

**Table 5:** Perioperative parameters and postoperative outcomes in the POCD and non-POCD groups.

	POCD	Non-POCD	*p*-value
Anesthesia duration	220.0 (185.0, 277.5)	215.0 (175.0, 270.0)	0.321
Intraoperative bleeding	575.0 (275.0, 1150.0)	500.0 (200.0, 850.0)	0.287
Fluid intake (ml)	1925.0 (1325.0, 2550.0)	1800.0 (1300.0, 2375.0)	0.412
ICU admission	7 (15.9%)	30 (7.8%)	0.128
Postoperative hospital day	7.0 (7.0, 9.0)	7.0 (5.0, 9.0)	0.320
*T*_Psi < 25_^*^	8.7 (0.3, 152.4)	0.8 (0.0, 51.3)	0.030
Incidence of delirium	23 (52.3%)	54 (14.1%)	<0.001
Duration of delirium	72.0 (48.0, 120.0)	24.0 (24.0, 72.0)	0.003
Subtype of delirium			0.081
hyper	6 (26.1%)	28 (51.9%)	
hypo	4 (17.4%)	9 (16.7%)	
mixed	13 (56.5%)	17 (31.5%)	

Values are presented as the median (Q1, Q3) or number of patients (%). POCD was defined as a decrease of three points compared with the baseline MMSE score at discharge.

* *T*_Psi < 25_ indicates the time at which Psi was less than 25. POCD, post-operative cognitive dysfunction; ICU, intensive care unit; MMSE, Mini Mental State Examination.

**Table 6:** Comparison of cognitive function and patient-centered quality of life test scores at baseline and at 1 week, 1 month, and 1 year after surgery between the POCD and non-POCD groups.

	POCD	Non-POCD	*p*-value
MMSE			
Baseline	27.0 (26.0, 28.0)	28.0 (26.0, 29.0)	0.025*
1 week	22.0 (19.5, 24.0)	28.0 (26.0, 29.0)	<0.001*
1 month	26.0 (24.0, 27.0)	29.0 (27.0, 29.0)	<0.001*
1 year	27.5 (27.0, 28.5)	29.0 (28.0, 30.0)	<0.001*
MoCA			
Baseline	22.0 (18.0, 24.0)	25.0 (22.0, 27.0)	<0.001*
1 week	17.5 (13.5, 22.0)	25.0 (22.0, 27.0)	<0.001*
1 month	23.0 (20.0, 24.0)	26.0 (23.0, 27.0)	<0.001*
1 year	25.0 (23.5, 28.0)	26.0 (24.0, 28.0)	0.028*
Modified version of the Telephone Interviews for Cognitive Status (TICS-m)			
1 month	32.5 (28.0, 33.0)	34.0 (31.0, 37.0)	0.127
1 year	29.1 ± 7.7	35.2 ± 6.0	0.001

Values are presented as mean ± SD or median (Q1, Q3).

* *p*-value for the *post hoc* analysis after the linear mixed model. POCD, postoperative cognitive dysfunction; MMSE, Mini Mental State Examination; MoCA, The Montreal Cognitive Assessment.

The findings of the univariate analysis revealed that POCD was associated with the Charlson Comorbidity Index, GDS scores, pre-existing MCI, duration of Psi < 25 in the operation, and preoperative benzodiazepine medication (Table 7). In the subsequent multivariate logistic regression analysis of preoperative variables, POCD was significantly associated with benzodiazepine medication, Charlson Comorbidity Index, pre-existing MCI, and GDS scores.

**Table 7:** Odds ratios on univariate and multivariate logistic regression analyses for POCD.

	Univariate model		Multivariate model (GOF test *p* = 0.999)*	
	Odds ratio (95% CI)	*p*-value	Odds ratio (95% CI)	*p*-value
Age (year)	1.07 (0.99–1.16)	0.070		
Education (<12 year)	1.78 (0.69–6.11)	0.287		
Old CVA	1.69 (0.55–4.30)	0.311		
Charlson comorbidity index (≥4)	2.80 (1.39–6.13)	0.006	2.88 (1.40–6.42)	0.006
Geriatric Depression Scale (≥4)	2.41 (1.28–4.70)	0.008	2.11 (1.08–4.22)	0.030
Pre-existing MCI	2.60 (1.31–5.02)	0.005	2.49 (1.22–4.96)	0.010
Anesthesia duration	1.00 (1.00–1.00)	0.317		
*T*_Psi < 25_	1.01 (1.00–1.01)	0.031		
Benzodiazepine medication	3.77 (1.56–8.52)	0.002	3.14 (1.24–7.45)	0.011
Postoperative urinary tract infection	2.83 (0.77–8.44)	0.081		

* Hosmer–Lemeshow goodness-of-fit test, *g* = 10. POCD, postoperative cognitive dysfunction.

GOF, goodness of fit; CI, confidence interval; CVA, cerebrovascular accident; MCI, mild cognitive impairment; *T*_Psi < 25_, time spent with a Psi value <25.

## 4 Discussion

In this study, benzodiazepine use, high comorbidity, pre-existing cognitive impairment, and disposition to depression were associated with POCD in elderly patients with no previous diagnosis of cognitive impairment. Cognitive screening, as part of the preoperative evaluation of elderly surgical patients, can assist in categorizing their risk levels, thereby enabling the implementation of suitable measures to mitigate negative postoperative outcomes. The administration of a cognitive test battery is essential if patients are at a higher risk of developing POCD (Tasbihgou and Absalom, 2021).

Similar to previous findings, our results also revealed that pre-existing MCI plays an important role in the development of POCD. The MoCA test meets the criteria for screening tests to detect MCI better than the MMSE (Langa and Levine, 2014; Elkana et al., 2020). Therefore, as per the criteria for MCI, we applied the age-adjusted cut-off value of the Korean version of the MoCA as different age groups may have different baseline cognitive performance and ability.

The TICS-m was used to evaluate cognitive function because conducting face-to-face cognitive testing on all enrolled patients was impossible due to the start of the coronavirus disease 2019 pandemic. Considering that the cut-off value of the TICS-m for diagnosing MCI is 24–25 (Seo et al., 2011; Pendlebury et al., 2013), there were significantly more patients with cognitive decline at 1 month and at 1 year after surgery (data not shown) in the MCI group, although we did not assess the baseline TICS-m score. It is well known that MCI is associated with postoperative adverse complications, such as postoperative delirium, mortality, readmission rate, and long hospital stay (Langa and Levine, 2014; Chen et al., 2022; Au et al., 2023). Nevertheless, in a practical clinical setting, preoperative evaluations of cognitive function are not routinely implemented. In this study, our findings revealed that even when patients with dementia and severe cognitive impairment were excluded, the prevalence of MCI was 19.9%, which has a serious impact on postoperative outcomes in elderly patients who have undergone surgery.

While postoperative delirium is usually of short duration, lasting 1–3 days, POCD can persist for several months and, sometimes, even longer (Liu et al., 2018). Postoperative delirium and POCD share several negative postoperative outcomes, such as longer hospital stays, higher mortality rates, and diminished quality of life (Langa and Levine, 2014). However, in the current study, the purpose of the multivariate logistic regression model was to identify risk factors for POCD. As we focused on the relationship between preoperative variables and POCD, postoperative delirium was not included in the multivariate logistic regression analysis.

In this study, although patients in the MCI group had higher GDS scores and higher scores in the depression domain of the PROMIS-29, they were not significantly higher. In other studies, a strong association has been observed between cognitive impairment and depression (Jung et al., 2013; Guan et al., 2020). Additionally, depression is associated with an increased likelihood of progression from MCI to dementia throughout the neurodegenerative continuum (Ismail et al., 2017). Interventions such as cognitive stimulation programs or cognitive training improve cognitive performance, anxiety, depression, and quality of life in elderly patients with MCI (Ge et al., 2018; Carcelén-Fraile et al., 2022). Further research is currently underway to determine whether applying cognitive stimulation programs or cognitive training in patients undergoing surgery is beneficial.

Using EEG-based anesthesia is controversial for the prevention of postoperative delirium or POCD (Punjasawadwong et al., 2018; Mahr et al., 2021). However, preventing very low levels of processed EEG monitoring is helpful in reducing the prevalence of postoperative delirium and POCD at 1 year after surgery (Mac Kenzie et al., 2018; Evered et al., 2021). In this study, the time of low Psi was higher in both the POCD and pre-existing MCI groups than in the other groups. It is uncertain whether EEG is causative of POCD or whether a low EEG score results from pre-existing MCI; however, there seems to be an association between intraoperative EEG and POCD.

Comorbidities such as hypertension, obesity, and diabetes mellitus are associated with cognitive decline in the general population (Needham et al., 2017). Our findings also showed that patients with a high degree of comorbidity had a high risk of developing POCD. However, according to other studies (Needham et al., 2017; Lertkovit et al., 2022), polypharmacy resulting from multiple comorbidities is not associated with POCD. Since benzodiazepines are associated with cognitive impairment (Gray et al., 2016), the relationship between benzodiazepines or sleep disturbances and POCD should also be considered. Our results also demonstrated that benzodiazepines could contribute to the development of POCD.

The aim of medicine has shifted from metrics of medical outcomes, such as mortality, to patient-reported outcomes and patient satisfaction (Fleisher, 2018). Modern anesthesia focuses on improving the quality of recovery after surgery. Postoperative pain control; biorhythms, such as sleep disturbances; and psychological recovery, such as depression and preoperative anxiety during surgery, are also important problems associated with returning to daily life after surgery. In the non-MCI group of this study, the PROMIS-29 score measured in various domains 1 month after surgery did not deteriorate compared to that before surgery. However, patients with pre-existing MCI reported worse postoperative PROMIS-29 scores than did patients without MCI in domains such as fatigue, anxiety, and depression. Patients screened for MCI should also be assessed for postoperative quality of recovery, and modifiable factors, such as insomnia or depression, should be corrected. Patients with POCD reported poor social roles and physical function on postoperative day 7. In high-risk elderly patients, follow-up of cognitive dysfunction and support for social and physical performance are imperative during the perioperative period.

This study has some limitations. First, this study has the inherent limitations of all observational cohort studies, such as various uncontrolled confounding factors and loss to follow-up. However, confounding and important risk factors could be distinguished using linear regression. To reduce the rate of loss during follow-up, we conducted telephone interviews using the TICS-m. Nevertheless, our study showed a significant loss to follow-up, especially in patients with poor condition who are admitted to a nursing hospital and so difficult to interview by phone. Second, neuropsychiatric cognitive battery tests were not conducted. However, the Korean versions of the MMSE, MoCA, and TICS-m have been validated and used in many other studies on MCI and POCD. These tests are convenient but time-consuming. Regrettably, in clinical settings, such a simple cognitive test is not implemented before surgery. The importance of this study is that the risk of POCD can be screened using these tests. Finally, since all participants in the study were Korean patients with no previous history of cognitive issues and had undergone spine surgery, it is necessary to conduct further research on other demographic groups to ensure that the present findings are applicable to the general population.

In conclusion, even after excluding severe cognitive impairment and dementia, preoperative MCI was seen in approximately 20% of surgical patients aged 70 years or older and was an important factor in POCD. POCD occurred in 1 out of 5 patients with pre-existing MCI and 1 out of 10 patients without pre-existing MCI. Benzodiazepine use, severe comorbidities, and depressive tendencies were risk factors for POCD.

## Data availability statement

The raw data supporting the conclusions of this article will be made available by the authors, without undue reservation.

## Ethics statement

The studies involving humans were approved by this was a secondary analysis of a previous prospective observational study that focused on postoperative delirium and was approved by the local institutional review board (Severance Hospital 4-2019-0654; ClinicalTrials.gov Identifier: NCT04120272). Written informed consent was obtained from all participants. The studies were conducted in accordance with the local legislation and institutional requirements. The participants provided their written informed consent to participate in this study.

## Author contributions

SP: Conceptualization, Data curation, Formal analysis, Resources, Visualization, Writing – original draft. JK: Conceptualization, Project administration, Writing – original draft. YH: Data curation, Resources, Software, Writing – original draft, Writing – review & editing. KK: Data curation, Resources, Software, Writing – review & editing. SY: Formal analysis, Validation, Writing – review & editing. B-NK: Conceptualization, Supervision, Writing – original draft, Writing – review & editing.
