# Supplementary material for: Preoperative mild cognitive impairment as a risk factor of postoperative cognitive dysfunction in elderly patients undergoing spine surgery
Source: Front Aging Neurosci. 2024 Jan 12;16:1292942. doi: 10.3389/fnagi.2024.1292942 (PMC10811182; doi:10.3389/fnagi.2024.1292942)
Supplement: Supplementary file 1 [file Data_Sheet_1.docx]

|  | | MCI | Non-MCI | P value | |
| --- | --- | --- | --- | --- | --- |
| PROMIS (Patient-Reported Outcomes Measurement Information System) -29, baseline | | | | | |
| Sleep Disturbance | 54.2 (43.9, 57.5) | | 51.1 (43.8, 56.2) | | 0.347 |
| Social Roles | 46.0 (40.9, 51.8) | | 48.0 (40.3, 55.5) | | 0.200 |
| Physical Function | 34.8 (32.5, 37.5) | | 36.2 (33.4, 40.3) | | 0.046 |
| Fatigue | 49.8 (40.0, 60.8) | | 46.0 (39.8, 55.2) | | 0.121 |
| Anxiety | 59.0 (44.1, 64.2) | | 53.0 (40.3, 61.4) | | 0.047 |
| Depression | 54.1 (41.0, 62.2) | | 51.6 (41.0, 57.1) | | 0.077 |
| PROMIS (Patient-Reported Outcomes Measurement Information System) -29, 1 week | | | | | |
| Sleep Disturbance | 52.9 (46.8, 58.1) | | 51.1 (46.4, 56.2) | | 0.205 |
| Social Roles | 40.2 (35.8, 48.1) | | 40.2 (35.8, 51.3) | | 0.630 |
| Physical Function | 34.0 (30.8, 36.3) | | 34.0 (30.8, 37.2) | | 0.837 |
| Fatigue | 53.2 (43.2, 62.2) | | 46.0 (39.8, 57.2) | | 0.014 |
| Anxiety | 55.6 (47.9, 67.4) | | 50.4 (40.3, 57.5) | | 0.005 |
| Depression | 51.6 (41.0, 58.9) | | 48.9 (41.0, 54.2) | | 0.014 |
| PROMIS (Patient-Reported Outcomes Measurement Information System) -29, 1 month | | | | | |
| Sleep Disturbance | 51.1 (47.2, 61.5) | | 47.2 (41.2, 54.5) | | 0.069 |
| Social Roles | 40.2 (38.1, 47.1) | | 48.0 (38.6, 55.5) | | 0.103 |
| Physical Function | 36.6 ± 5.6 | | 37.7 ± 6.2 | | 0.417 |
| Fatigue | 48.7 (46.0, 55.2) | | 46.0 (39.8, 54.5) | | 0.349 |
| Anxiety | 55.3 (47.9, 63.1) | | 47.9 (40.3, 56.0) | | 0.039 |
| Depression | 48.9 (41.0, 55.9) | | 41.0 (41.0, 54.1) | | 0.128 |

Supplementary table 1. patient-centered quality of life at baseline, and one week and one month after surgery between the MCI and non-MCI groups

|  | | POCD | Non-POCD | | P value |
| --- | --- | --- | --- | --- | --- |
| PROMIS (Patient-Reported Outcomes Measurement Information System) -29, baseline | | | | | |
| Sleep Disturbance | 57.5 (44.2, 61.9) | | | 51.1 (43.8, 55.5) | 0.049 |
| Social Roles | 44.2 (38.6, 55.5) | | | 48.0 (41.2, 55.5) | 0.422 |
| Physical Function | 34.4 (32.1, 40.2) | | | 35.6 (33.4, 40.2) | 0.118 |
| Fatigue | 53.9 (43.1, 62.7) | | | 46.0 (39.8, 55.2) | 0.095 |
| Anxiety | 63.5 (40.3, 67.4) | | | 53.6 (40.3, 61.4) | 0.091 |
| Depression | 54.3 (41.0, 64.0) | | | 52.0 (41.0, 57.3) | 0.292 |
| PROMIS (Patient-Reported Outcomes Measurement Information System) -29, 1 week | | | | | |
| Sleep Disturbance | 54.5 (49.0, 60.6) | | | 51.1 (46.4, 56.2) | 0.053 |
| Social Roles | 37.2 (31.8, 42.3) | | | 40.2 (35.8, 50.7) | 0.023 |
| Physical Function | 31.3 (28.7, 35.3) | | | 34.0 (30.8, 37.2) | 0.046 |
| Fatigue | 55.2 (43.1, 61.6) | | | 48.2 (39.8, 58.9) | 0.161 |
| Anxiety | 55.3 (47.9, 61.4) | | | 51.1 (40.3, 57.7) | 0.053 |
| Depression | 51.2 (48.9, 59.6) | | | 48.9 (41.0, 54.7) | 0.051 |
| PROMIS (Patient-Reported Outcomes Measurement Information System) -29, 1 month | | | | | |
| Sleep Disturbance | 50.5 (41.2, 61.9) | | | 49.0 (41.2, 54.5) | 0.694 |
| Social Roles | 46.1 (33.9, 55.5) | | | 47.1 (38.6, 55.5) | 0.739 |
| Physical Function | 35.8 ± 7.7 | | | 37.7 ± 6.0 | 0.354 |
| Fatigue | 48.6 (39.8, 51.2) | | | 46.0 (39.8, 55.2) | 0.793 |
| Anxiety | 53.7 (40.3, 66.8) | | | 47.9 (40.3, 57.2) | 0.412 |
| Depression | 45.0 (41.0, 55.9) | | | 48.9 (41.0, 54.1) | 0.938 |

Supplementary table 2. patient-centered quality of life at baseline, and one week and one month after surgery between the POCD and non-POCD groups
